# Two-Photon Time-Gated In Vivo Imaging of Dihydrolipoic-Acid-Decorated Gold Nanoclusters

**DOI:** 10.3390/ma14247744

**Published:** 2021-12-15

**Authors:** Ye Tian, Ming Wei, Lijun Wang, Yuankai Hong, Dan Luo, Yinlin Sha

**Affiliations:** 1 Department of Biophysics, Single-Molecule and Nanobiology Laboratory, School of Basic Medical Sciences, Peking University, Beijing 100191, China; weiming@bjmu.edu.cn (M.W.); lijunwang@bjmu.edu.cn (L.W.); 2 CAS Center for Excellence in Nanoscience, Beijing Key Laboratory of Micro-Nano Energy and Sensor, Beijing Institute of Nanoenergy and Nanosystems, Chinese Academy of Sciences, Beijing 100083, China

**Keywords:** two-photon imaging, time-gated imaging, gold nanoclusters, in vivo imaging

## Abstract

Due to the unique advantages of two-photon technology and time-resolved imaging technology in the biomedical field, attention has been paid to them. Gold clusters possess excellent physicochemical properties and low biotoxicity, which make them greatly advantageous in biological imaging, especially for in vivo animal imaging. A gold nanocluster was coupled with dihydrolipoic acid to obtain a functionalized nanoprobe; the material displayed significant features, including a large two-photon absorption cross-section (up to 1.59 × 10^5^ GM) and prolonged fluorescence lifetime (>300 ns). The two-photon and time-resolution techniques were used to perform cell imaging and in vivo imaging.

## 1. Introduction

The imaging of living animals is of extreme importance in the study of biotic changes and for the valuable information provided by disease diagnoses and health assessments at different levels, such as organs, tissues, etc. Among the many modalities of imaging, non-invasive and in vivo imaging are especially significant in studying long-term animal models.

In a broad sense, imaging modalities can be classified into morphological and molecular imaging techniques. In practical terms, magnetic resonance imaging (MRI), computed tomography (CT), and high-frequency micro-ultrasound are often used for anatomical imaging, and for molecular visualizations, optical imaging (including fluorescence imaging and bioluminescence imaging), positron emission tomography (PET), and single-photon-emission computed tomography (SPECT) are used [1].

In comparison with other methods of non-invasive and in vivo imaging, such as CT, MRI, PET, etc., in vivo fluorescence imaging has some advantages, such as its high sensitivity, fast and easy control, multi-targeting capabilities, fast imaging, high stability, low toxicity, non-ionizing radiation, low cost, etc. [2,3], and there have been many studies relating to it, including studies of the optimization of the parameters and in vivo imaging of different tissues [4,5].

With the emergence of the femtosecond laser, two-photon fluorescence imaging rapidly developed. It has additionally gained more advantages than one-photon fluorescence imaging. For conventional fluorescence dyes, the two-photon excitation is within the red to near-infrared (NIR) region. The photo-damage to the biological samples is smaller in this region, in which the absorption of biological chromophores (such as hemoglobin) and H_2_O is decreased, the autofluorescence is diminished, and the scatter of light in the tissue is reduced, so two-photon imaging displays a higher signal-to-noise ratio and deeper penetration into the tissue (>500 μm) [6,7]. Because the laser beam of two-photon excitation is focused at a focal point, the potential spatial resolution is improved, and photo-bleaching is reduced [8].

In addition to two-photon fluorescence imaging, another approach to improving the signal-to-noise ratio is to carry out time-gated imaging, which is based on the distinctions of the fluorescence lifetime. This strategy is used to temporally distinguish the background and signals, thereby minimizing the impact of autofluorescence on imaging quality.

However, the two-photon absorption (TPA) cross-sections of traditional organic dyes are small [9], and there is little difference in the fluorescence lifetime between traditional organic dyes and biological tissues [10,11], which affects the imaging quality. Therefore, fluorescence probes with larger TPA cross-sections and longer fluorescence lifetimes are required. Moreover, other characteristics, such as low photo-bleaching, high quantum yield, the no-blinking phenomenon, non-toxicity to organisms, and ease of synthesis and application are also required for the ideal probes, and the size of the fluorescence probes should be small enough so that the normal function of biomolecules will not be affected.

Gold nanoclusters (AuNCs) were extensively studied in many research fields, such as those of catalysts, bioassays, and fluorescence imaging. AuNCs are made of several to hundreds of atoms, so the size is smaller than 2.2 nm. AuNCs exhibit photoluminescence (PL) ranging from the visible to NIR spectra [12], and the two-photon-induced fluorescence of AuNCs was studied, indicating that AuNCs display large TPA cross-sections [13,14]. The fluorescence lifetime of AuNCs is remarkably long (>100 ns), while the fluorescence lifetime of autofluorescence and most organic dyes is short; thus, the long fluorescence lifetime of AuNCs can be used to improve the imaging contrast [15]. In addition, AuNCs display a small size, reduced biological toxicity, and a large Stokes shift, which are beneficial for biological imaging [16].

In this work, dihydrolipoic acid (DHLA)-protected AuNCs (DHLA-AuNCs) with red to NIR emission spectra were synthesized and conjugated to galactosamine, which can recognize hepatoma through asialoglycoprotein receptor (ASGPR). Moreover, the advantages of the strong two-photon absorption and long fluorescence lifetime of DHLA-AuNCs are simultaneously shown. With a new device combining the imaging technologies of two-photon excitation and time-gated imaging, in vivo two-photon time-gated imaging was performed with tumor-bearing animal models.

## 2. Materials and Methods

### 2.1. The Chemicals

All chemicals were used directly without further purification: chloroauric acid (HAuCl_4_, Shanghai Jingchun Industrial Co., Shanghai, China), sodium borohydride (NaBH_4_, 98%, Shanghai Jingchun Industrial Co. Shanghai, China), reduced glutathione (GSH, 98%, Beijing Biodee Biotech. Co. Beijing, China), Dulbecco’s Modified Eagle’s Medium (DMEM, Invitrogen, California, USA), fetal bovine serum (FBS, 10%, Beijing Xinjingke Biotech. Co. Beijing, China), penicillin/streptomycin (1%, Beijing Xinjingke Biotech. Co. Beijing, China), trypsin (0.25%, Hyclone, California, CA, USA), (4,5-Dimethylthiazol-2-yl)-2,5-diphenyltetrazolium bromide (MTT, 98%, Amresco, California, CA, USA), dimethyl sulfoxide (DMSO, 99%, Sigma-Aldrich, Darmstadt, Germany), Rhodamine B (95%, Sigma-Aldrich, Darmstadt, Germany), D-(+)-Galactosamine hydrochloride (99%, Shanghai Jingchun Industrial Co., Shanghai, China), N-(3-Dimethylaminopropyl)-N’-ethylcarbodiimide hydrochloride (EDC, 98.5%, Shanghai Jingchun Industrial Co., Shanghai, China), methoxypolyethylene glycol amine (Mw: 2000, Shanghai Jingchun Industrial Co., Shanghai, China), and isoflurane (1.5%, Shandong Keyuan Pharmaceutical Co., Shandong, China). Ultrapure water (18.2 MΩ cm ^− 1^) was used in the experiments.

### 2.2. Synthesis of DHLA-AuNCs

The method of synthesizing DHLA-AuNCs employed here had a slight change compared with that reported previously [17,18]. DHLA (11.5 μL) was briefly added to a methanol solution (5 mL) of HAuCl_4_ (0.005 M) and stirred for half an hour in a cool bath at 0 °C. Then, a 0 °C NaBH_4_ (1.25 mL, 0.16 M) aqueous solution was rapidly added to the mixture under vigorous stirring, and the reaction continued for another hour. The product was precipitated by adding HCl (1.5 mL, 1 M) and centrifuged for 10 min at 12,000 rpm. After that, the supernatant was removed, and the precipitates were dissolved with water (12 mL); the pH value was adjusted to 9.0 with NaOH (1 M). Then, DHLA (9.2 μL) was added, and the pH value of the solution was adjusted to 9.0. The etching process lasted for an hour under stirring at 55 °C in a water bath. After applying ultrafiltration and centrifugation thrice, the DHLA-AuNCs were obtained for the following characterization and application.

### 2.3. Characterization of DHLA-AuNCs

The emission and excitation spectra were assessed on an F-4500 fluorescence spectrophotometer (Hitachi) equipped with a temperature controller. The excitation and emission wavelengths were set at 400 and 617 nm. A quartz cuvette with a path length of 10 mm was used. The concentration of DHLA-AuNCs in all of the tests was consistent (8.62 μM). The absorption spectra were acquired with a TU-1901 UV–vis spectrophotometer.

The PL spectra were recorded at various pH levels to evaluate the pH-relevant photo-stability of the DHLA-AuNCs. Concretely, the pH of the DHLA-AuNCs was adjusted from 4.0 to 9.0 with HCl or NaOH while keeping the final concentration of DHLA-AuNCs the same. The temperature of the tests was kept at 20 °C, and the PL measurements were repeated thrice at each pH point.

In order to assess the resistance of the reduced glutathione, the DHLA-AuNCs were incubated with reduced glutathione (2 μm) in phosphate-buffered saline (PBS, 10 mM, pH 7.4) at 37 °C, and the PL intensity was recorded at different time points (1, 15, 30, 60, 120, 180, and 300 min) to reflect the resistance.

The photo-bleaching resistance tests were performed on the F-4500 fluorescence spectrophotometer while the temperature was kept at 20 °C; the excitation and emission wavelengths were set to 400 and 617 nm, and the tests lasted for 1 h.

The fluorescence decay of the DHLA-AuNCs in H_2_O was measured with a LifeSpec Red Spectrometer (Edinburgh Inc., Edinburgh, UK.) with an excitation wavelength of 372 nm.

Transmission electron microscopy (TEM) was performed on an FEI Tecnai F20 transmission electron microscope operating at 200 kV.

The hydrodynamic diameters of the DHLA-AuNCs were measured on a NICOMPTM380ZLS system (PSS, California, CA, USA). The zeta potential was measured at 25 °C on a Zeta Plus Potential Analyzer (Brookhaven, NY, USA). The DHLA-AuNCs were dispersed in PBS (the concentration of DHLA-AuNCs was 8.62 μM). A particle size distribution analysis was conducted by using NICOMP number-weighted distribution analysis.

Cytotoxicity tests were performed according to the ATCC MTT cell proliferation assay protocol. Briefly, HepG2 and Hela cells were cultured and passaged in Dulbecco’s Modified Eagle’s Medium (DMEM, Invitrogen, California, CA, USA) with fetal bovine serum (FBS, 10%) and penicillin/streptomycin (1%). HepG2 and Hela cells were detached with trypsin (0.25%) and suspended in DMEM containing 10% FBS and 1% penicillin/streptomycin; the cells were seeded at a density of 5 × 10^3^ cell/well in 96-well plates, and after incubation for 24 h at 37 °C with 5% CO_2_, different amounts of DHLA-AuNCs were added to each well, reaching final DHLA-AuNC concentrations of 0, 0.035, 0.086, 0.17, 0.35, 0.86, 1.72, 3.45, and 8.62 μM. After another 24 h of incubation, the supernatant was removed, and each well was washed thrice with PBS, then replenished with DMEM. Then, 3-(4,5-Dimethylthiazol-2-yl)-2,5-diphenyltetrazolium bromide (MTT, Amresco, 10 μL, 5 mg mL^−1^) was added to each well. After incubation for 4 h, the culture medium was discarded, and dimethyl sulfoxide (Sigma-Aldrich, 100 μL) was added to dissolve the newly formed water-insoluble formazan blue intracellularly. The absorbance was measured at 570 nm on a BioRad model 550 microplate reader. The data analysis was conducted by using Student’s t-test, and differences with *p*-values <0.05 were considered to be statistically significant.

The thermal properties of the DHLA-AuNCs were studied using a thermoanalyzer (model Q 600 SDT (TA Co.)) at a constant heating rate of 10 °C/min from 25 to 700 °C under a nitrogen atmosphere.

The two-photon absorption (TPA) cross-section of the DHLA-AuNCs was studied by using the method of two-photon-induced fluorescence with Rhodamine B as a standard reference [19].

The laser pulse was generated with a mode-locked Ti:Sapphire laser (730–860 nm, ≈80 fs, 82 MHz, Tsunami, Spectra Physics) focused with a quartz cuvette with a 10 mm optical path length. A variable neutral-density filter was applied to adjust the intensity of the incident laser beam. To detect the two-photon-induced fluorescence, a liquid-nitrogen-cooled charge-coupled device (CCD) detector (SPEC-10-400B/LbN, Roper Scientific, California, USA) attached to a polychromator (Spectropro-550i, Acton, California, CA, USA) was used.

The DHLA-AuNCs were dissolved in ddH_2_O (10^−4^ M), while the reference Rhodamine B was dissolved in methanol at the same concentration.

### 2.4. Synthesis and Characterization of Gal-DHLA-AuNCs

To synthesize the probes for the in vitro and in vivo tests, DHLA-AuNCs (8.6 × 10^−3^ μmol) and D-(+)-Galactosamine hydrochloride (0.95 μmol) were dissolved and mixed in ddH_2_O; then, N-(3-Dimethylaminopropyl)-N′-ethylcarbodiimide hydrochloride (EDC) (1 μmol) was added, and the mixture above was left at 20 °C to continue the reaction for 1 min. Then, methoxypolyethylene glycol amine (0.05 μmol) was added and was allowed to continue to react for 2 h, and the products were noted as Gal-DHLA-AuNCs.

The free ingredients were removed using centrifugal filter devices (Amicon Ultra-4, Millipore, Darmstadt, Germany), and the probes were lyophilized and stored at 4 °C. Fourier transform infrared (FTIR) spectroscopy was conducted to confirm the conjugation.

The Fourier transform infrared (FTIR) spectra were acquired on a NEXUS-470 FTIR spectrometer (Nicolet, Wisconsin, WI, USA) using KBr pellets in the range from 4000 to 400 cm^−1^. Samples were lyophilized using a freeze-drier (Alpha 1-4, Christ, Osterode, Germany). Data were collected at a resolution of 8 cm^−1^ with 256 scans.

### 2.5. In Vitro Two-Photon Imaging

In order to perform the in vitro imaging, HepG2 cells were detached and seeded on a Petri dish (10 mm). After another 12 h of culture to allow the cells to attach, the cells were incubated in PBS with Gal-DHLA-AuNCs (0.34 μM) for 2 h at 37 °C; then, the cells were rinsed thrice with PBS for fluorescence imaging. One-photon excitation experiments were performed on a Zeiss Axio ObserverZ1 optical system (Zeiss, Jena, Germany), which irradiated with a 488 nm laser (3.0 W cm^−2^). Two-photon excitation experiments were performed on a Zeiss LSM 780 NLO optical system (Zeiss, Jena, Germany) equipped with a tunable Ti:sapphire laser (Mai Tai HP DS, Newport, RI, USA), which irradiated with an 800 nm laser (13.8 W cm^−2^).

### 2.6. In Vivo Two-Photon Time-Gated Imaging

Male BALB/c nude mice were purchased from the Experimental Animal Center of Peking University Health Science Center at 6 weeks of age (initially weighing 20–25 g). All animal procedures were approved by the Committee on Ethics of Beijing Institue of Nanoenergy and Nanosystems (No. A-2019003).

In order to establish a solid tumor-bearing mice model, HepG2 cells were cultured, detached, and re-suspended in PBS (4 × 10^6^ mL^−1^). The suspension (100 μL) was subcutaneously inoculated at the right thigh. When the tumors reached 150–200 mm^3^ in volume, Gal-DHLA-AuNCs dispersed in PBS were intravenously administered to mice via the tail veins (0.1 mL (86.2 μM)/mouse (0.8 nmol g^−1^ of body weight in reference [20])). The tumor volumes were calculated with the following equation:

(1) $$V=\mathrm{length}\;(\mathrm{cm})\times {\mathrm{width}}^{2}{\;(\mathrm{cm}}^{2})\times 0.5236$$

The mice were anesthetized with isoflurane (1.5%), and two-photon time-gated images were scanned at different time points (5, 30, and 60 min) after the administration. Then, the mice were sacrificed, the tumors, liver, and kidneys were collected, and the tissues were immediately scanned with the same conditions as in vivo.

For the two-photon time-gated imaging, the mean power of laser excitation at 800 nm was 500 mW, and the beam was 12 mm in diameter, with a Gaussian distribution of the energy density, a regenerative amplifier (Spitfire, Spectra Physics, 100 fs, 500 Hz) running at the wavelength of 800 nm was used to provide the excitation pulses. No focusing adjustment for the excitation laser beam was implemented until it was used on the samples. A set of lenses was employed to collect the luminescence signals from the samples. The signal was sent to an ICCD detector (PI-MAX 1024 Unigen III Princeton Instruments, NJ, USA). In order to remove the unnecessary light, a bandpass filter (FF01-615/24, Semrock) was used in the lens set. The ICCD gates were manipulated by the inner delay of the ICCD controller, and the delay time between the femtosecond laser pulses was set to 500 ns to allow the full decay of autofluorescence (the autofluorescence decayed within 100 ns) [21,22].

The arrangement of the devices is shown in Figure S1 of the Supplementary Materials; panel (A) represents the working laser pulses, the orientation of which was fixed, and to scan the regions of interest, a moveable object stage (B) was employed. After scanning and recording the signals of the region of interest (the area of which was 1.28 cm × 1.28 cm), the object stage was translationally moved to another region of interest; panel (C) presents the inhalation mask for anesthesia.

## 3. Results and Discussion

### 3.1. Characterization of DHLA-AuNCs

**Figure 1:**
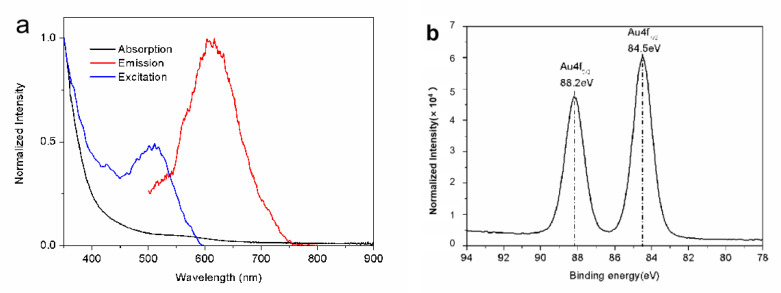
(**a**) The typical spectra of the DHLA-AuNCs. (**b**) The XPS spectrum shows the binding energy of the Au4f level of the DHLA-AuNCs.

Figure 1a shows the typical emission and excitation spectra of the dihydrolipoic acid (DHLA)-stabilized AuNCs (DHLA-AuNCs). The emission spectrum was centered at 617 nm, and a full width at half maxima (FWHM) of 106 nm was observed in ddH_2_O upon excitation at 400 nm. The emission range of the DHLA-AuNCs is beneficial for biological imaging because, in biological samples, the absorption of light in the red to NIR range is reduced compared with that in the UV and infrared regions, and a longer wavelength of the emitted light also reduces the scatter in the tissue; these factors induce deeper penetration [23].

The quantum yield of the DHLA-AuNCs was determined to be 0.14% in H_2_O (pH 9.0), with Rhodamine 6G (QY = 0.95 in ethanol) as a reference. The PL emissions of the AuNCs were strongly affected by ligands; it was reported that the ligands containing electron-rich atoms (e.g., O, N, etc.) or groups improve PL emissions greatly [24,25]. By applying ligands containing electron-rich atoms or groups, AuNCs with a high quantum yield were reported [26], and the quantum yield of DHLA-AuNCs was reported to be 1.83% in H_2_O [27].

The absorption spectrum is shown in Figure 1a; unlike large gold nanoparticles, DHLA-AuNCs show one weak absorption band centered at 560 nm, rather than a strong surface plasmon resonance. This absorption band was believed to be derived from metal-centered and/or ligand-to-metal charge transfer transitions [28].

X-ray photoelectron spectroscopy (XPS) measurements were carried out to analyze the valence states of gold in DHLA-AuNCs; the XPS spectrum (Figure 1b) shows the binding energy (BE) of Au 4f_5/2_ and Au4f_7/2_ at 88.2 and 84.5 eV, respectively. The BE of Au4f_7/2_ fell between that of the Au(0) BE (83.8 eV) of a metallic gold film and the Au(Ⅰ) BE (86 eV) of gold thiolate, suggesting the coexistence of Au(0) and Au(Ⅰ) in the DHLA-AuNCs [27].

Bidentate thiolate-protected AuNCs [29,30] (e.g., with DHLA) were studied; the chemical bonds between the surface atoms of the core and the ligands are stronger, which brings about the extremely high stability of AuNCs [31]. In addition, the carboxyl groups of DHLA provide anchors for immobilizing biological molecules [32].

In order to study the feasibility of applying DHLA-AuNCs to biological systems, the stability of the DHLA-AuNCs was studied.

The PL intensity of the DHLA-AuNCs manifested a slight change when the pH was adjusted between 4.0 and 9.0 (Supplementary Figure S2), and alkaline conditions were optimal for PL emissions (pH 9). These phenomena can be associated with the pKa of DHLA. The pKa of DHLA is 4.9; alkaline conditions improve the solubility of DHLA and, thus, the PL emissions of DHLA-AuNCs.

It was acknowledged that reduced glutathione (GSH) is abundant in living organisms, as the reduced glutathione–oxidized glutathione (GSH-GSSG) equilibrium plays a significant role in protecting organisms from free radical damage and oxidative stress [33,34]. Since the thiols of GSH may react with DHLA-AuNCs, the resistance of DHLA-AuNCs against reduced glutathione was checked in the case of instability in the systemic circulation.

The DHLA-AuNCs were incubated in phosphate-buffered saline (PBS, pH 7.4) with GSH (2 μm, the physiological concentration in human plasma [35]) to reflect the resistance of GSH (Supplementary Figure S3). The PL intensity remained stable in the following incubation for 5 h, implying the good resistance of the reduced glutathione in the blood and, thus, the prospect of applying DHLA-AuNCs in vivo.

In order to estimate the anti-photobleaching property of DHLA-AuNCs, the DHLA-AuNCs were continuously exposed to 400 nm light for 1 h. No decrease in PL intensity was observed (Supplementary Figure S4), indicating the good anti-photobleaching properties of the DHLA-AuNCs.

**Figure 2:**
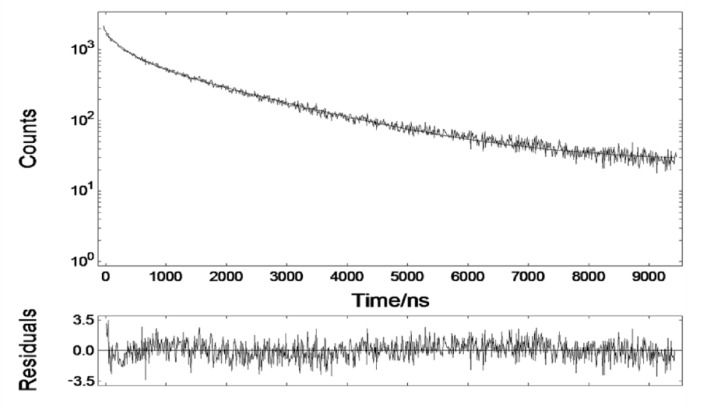
The fluorescence decay of DHLA-AuNCs in aqueous solution.

The fluorescence of the DHLA-AuNCs exhibited a double-exponential decay in H_2_O (321.24 ns (16.27%), 1808.17 ns (83.73%)) (Figure 2). The microsecond emission was deemed to be derived from S–Au hybrid states [36].

The fluorescence lifetime of DHLA-AuNCs was much longer than that of cellular autofluorescence and most organic dyes. This tremendous disparity acts as a prerequisite for the minimization of the influence of autofluorescence in fluorescence imaging and, thus, for the improvement of the signal-to-noise ratio, which makes DHLA-AuNCs promising biomarkers for in vivo time-gated imaging.

**Figure 3:**
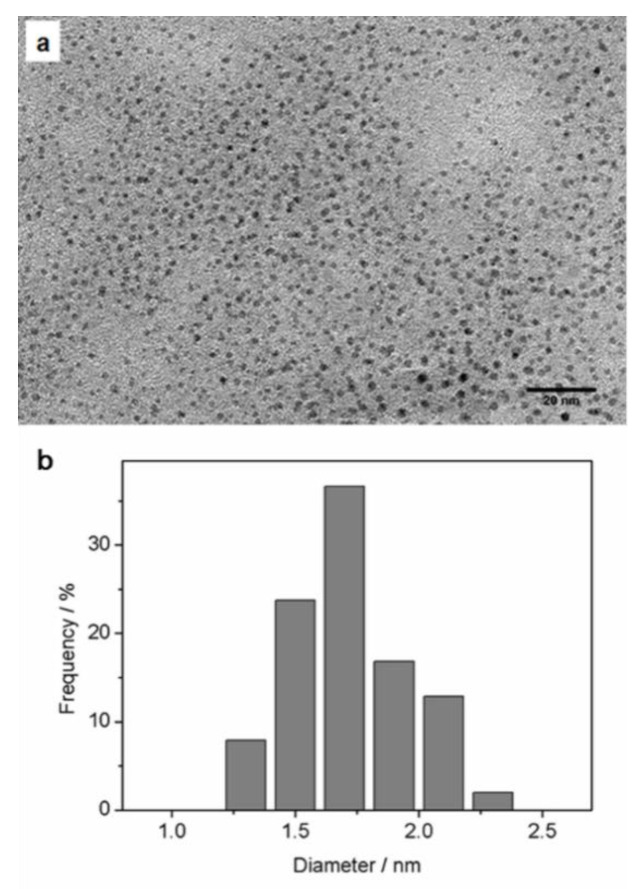
(**a**) A representative TEM image of DHLA-AuNCs revealing their cores; (**b**) the corresponding size distribution; the average diameter was 1. 71 nm ± 0.233 nm.

Transmission electron microscopy (TEM) revealed the cores of the DHLA-AuNCs (Figure 3a), and the average diameter was 1.71 ± 0.23 nm (from the imaging analysis of more than 100 random individual particles) (Figure 3b).

The hydrodynamic diameter was determined by dynamic light scattering (DLS) to be 3.7 ± 0.54 nm (Supplementary Figure S5); the small size of less than 5 nm makes DHLA-AuNCs attractive as fluorescent probes since the tiny dimension minimizes the influence on biological processes and provides more meticulous images with an improved resolution [37,38]. The zeta potential of the DHLA-AuNCs dispersed in pure water (pH 7) was measured to be –20.3 mV.

**Figure 4:**
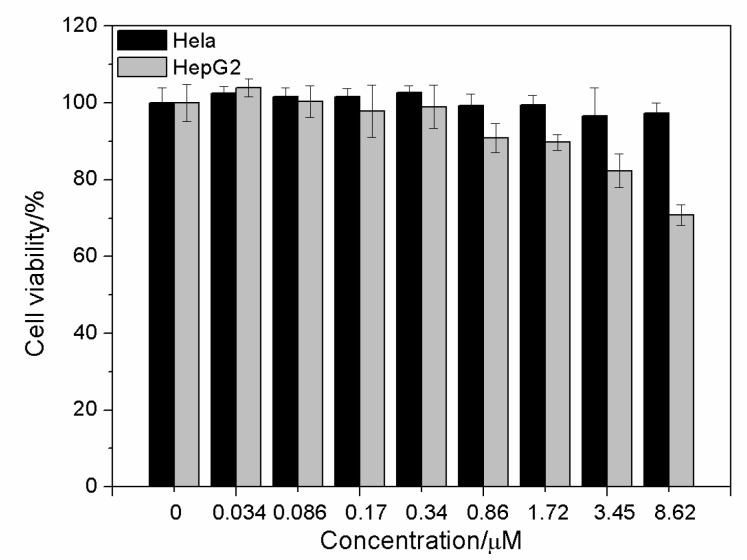
Cytotoxicity of DHLA-AuNCs at various concentrations after incubation with Hela cells and HepG2 cells for 24 h.

The DHLA-AuNCs were of low cellular toxicity; after being incubated with DHLA-AuNCs for 24 h, the viability of Hela cells remained above 90% at the concentration of 8.62 μM; for HepG2 cells, the viability was also around 80% at the concentration of 3.45 μM (Figure 4).

The innocuousness of DHLA-AuNCs is promising for potential applications in imaging in vitro and in vivo.

In order to study the two-photon absorption cross-section of the DHLA-AuNCs, it was necessary to decide on the average weight of the DHLA-AuNCs. TEM and thermo-gravimetric analysis (TGA) were implemented to accomplish this mission.

The average number of gold atoms in each DHLA-AuNC was calculated to be 154 according to the TEM analysis (details are shown in the Supplementary Materials).

TGA revealed the percentage of the ligands to be 47.75% (Figure S6). The formula was determined to be Au_154_DHLA_134_, and the average molecular weight of the DHLA-AuNCs was calculated to be 5.81 × 10^4^ g mol^−1^. According to the Lambert–Beer law (A = εbC), the molar extinction coefficient was determined to be 1.06 × 10^4^ at 470 nm.

The two-photon absorption (TPA) cross-section (δ) of the DHLA-AuNCs was studied by using the method of two-photon-induced fluorescence, using Rhodamine B as a standard reference [19].

**Figure 5:**
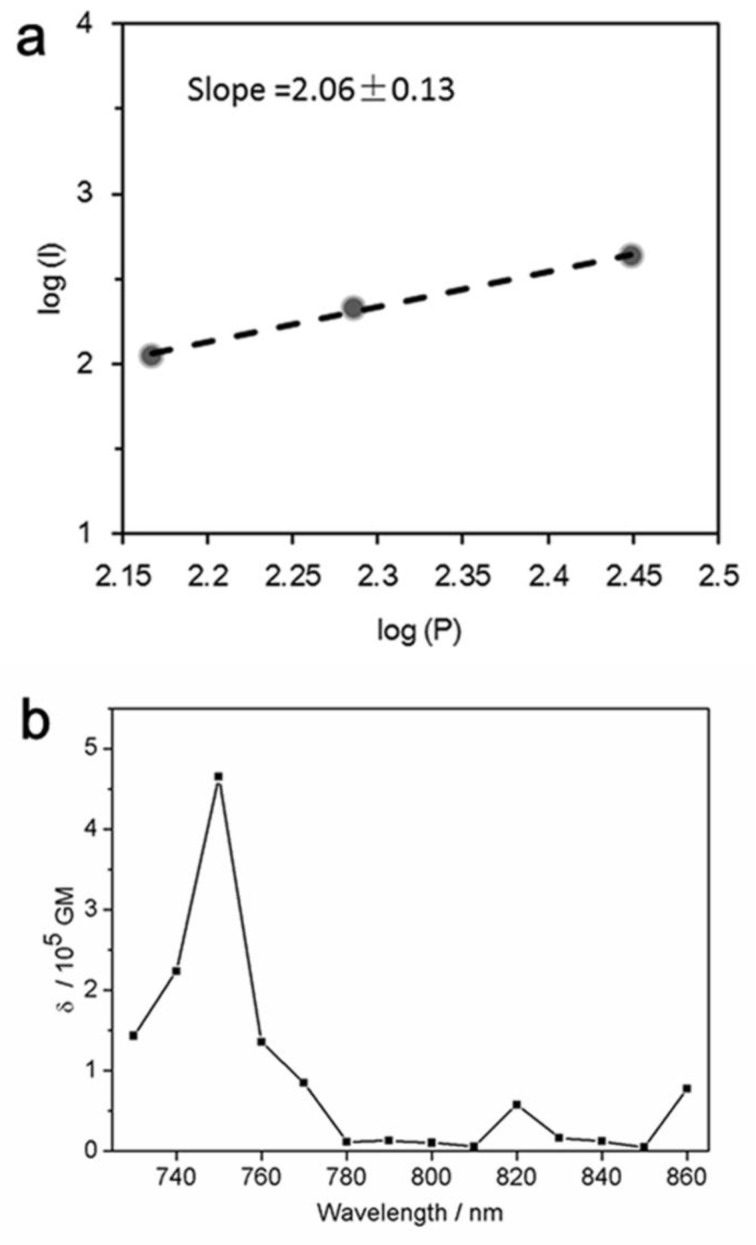
(**a**) The quadratic dependence of the two-photon emission intensity on the excitation power with two-photon excitation at 750 nm; (**b**) two-photon absorption cross-section of the DHLA-AuNCs as a function of wavelength.

The quadratic dependence of the two-photon emission intensity on the excitation power (Figure 5a) confirmed the occurrence of two-photon excitation.

As shown in Figure 5b, the maximal δ of the DHLA-AuNCs was 1.59 × 10^5^ GM at 750 nm (Göppert–Mayer (GM) units (10^−50^ cm^4^ s photon^−1^)).

The two-photon absorption (TPA) cross-section (δ) is the basic parameter in evaluating the TPA properties, e.g., the probability of a molecule absorbing two photons simultaneously. The gold nanoclusters were reported to display much larger TPA cross-sections than those of traditional organic dyes and quantum dots [39,40], and it was believed that the large TPA was caused by the free electrons of AuNCs and the polarizability aroused by the free movement of electrons in the nanoclusters [41,42].

### 3.2. In Vitro Two-Photon Imaging of DHLA-AuNCs

Galactose-based materials were extensively used to detect hepatocellular carcinomas (HCCs). When galactose is specifically bound to the asialoglycoprotein receptor (ASGPR), which is widely expressed in hepatic parenchymal cells and hepatocellular carcinoma cells (e.g., the HepG2 cell line) [43,44], applications of galactose-based anti-tumor drugs tend to limit the side effects on the hepatic system.

In order to achieve galactose-based tumor imaging, D-(+)-Galactosamine hydrochloride and methoxypolyethylene glycol amine were conjugated to DHLA-AuNCs using carbodiimide as a zero-length cross-linker to generate galactose-linked DHLA-AuNCs (Gal-DHLA-AuNCs). The Fourier transform infrared (FTIR) spectra of the conjugated probe Gal-DHLA-AuNCs and DHLA-AuNCs are shown in Supplementary Figure S7.

HepG2 cells that express high levels of ASGPR were incubated with the Gal-DHLA-AuNCs for 2 h at 37 °C, and both one-photon fluorescence imaging and two-photon fluorescence imaging were carried out.

**Figure 6:**
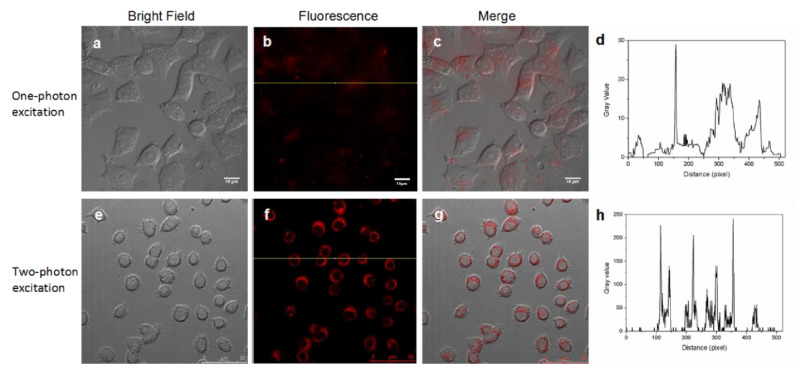
DHLA-AuNCs are competent probes in both one-photon excitation (the upper panels) and two-photon excitation (the lower panels) fluorescence imaging. (**a**) The bright field image with one-photon excitation; (**b**) The fluorescence image with one-photon excitation; (**c**) The merged image of (**a**) and (**b**); (**e**) The bright field image with two-photon excitation; (**f**) The fluorescence image with two-photon excitation, (**g**) The merged image of (**e**) and (**f**); (**d**,**h**) The gray value profiles of the cross-lines in (**b**) and (**f**).

The upper panels of Figure 6 show the results of the one-photon excitation imaging performed on a Zeiss Axio ObserverZ1 optical system when irradiated with a 488 nm laser (3.0 W cm^−2^), while the lower panels present the two-photon excitation results performed on a Zeiss LSM 780 NLO optical system equipped with a tunable Ti:sapphire laser (Mai Tai HP DS) when irradiated with an 800 nm laser (13.8 W cm^−2^). Figure 6a,e present differential interference contrast (DIC) images of HepG2 cells without obvious morphological changes, indicating the few toxic effects of the DHLA-AuNCs on the living cells, and strong red two-photon excitation signals were identified in the HepG2 cells (Figure 6b,f) with high efficiency of detection (Figure 6c,g). More importantly, these studies proved that DHLA-AuNCs could be competently applied to both one-photon and two-photon fluorescence imaging.

Figure 6d shows the signal profile along the line in Figure 6b, while the signal profile along the line in Figure 6f is shown in Figure 6h. This comparison exhibits the contrast between the signal and background in both imaging patterns, showing the better discrimination of the signal from the background in two-photon fluorescence imaging.

Other than the superior signal-to-noise ratio, two-photon fluorescence imaging still possesses other advantages, including the augmented penetration depth of excitation light, increased fluorescence collection efficiency, and reduced phototoxicity, all of which make two-photon fluorescence imaging more advantageous than single-photon fluorescence microscopy [45].

### 3.3. In Vivo Two-Photon Time-Gated Imaging of DHLA-AuNCs

**Figure 7:**
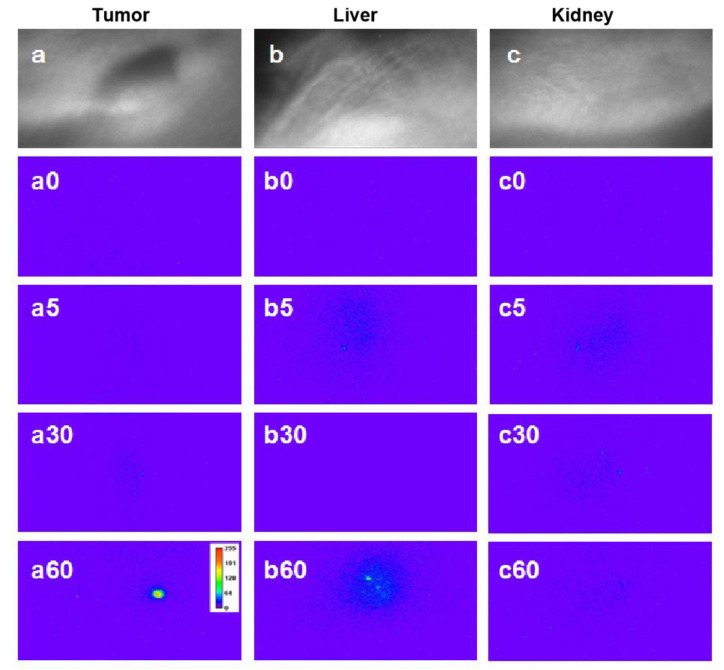
The regional bright-field images of three tissues: (**a**) tumor, (**b**) liver, and (**c**) kidney. Panels (**a0**–**c0**) show the respective two-photon time-gated images of the three tissues before the administration of Gal-DHLA-AuNCs; the numbers after the letters stand for the time after administration. Panels (**a5**), (**a30**), and (**a60**) display the regional two-photon time-gated images of the tumor at different time points (5, 30, and 60 min). Panels (**b5**), (**b30**), and (**b60**) displayed the regional two-photon time-gated images of the liver at different time points (5, 30, and 60 min). Panels (**c5**), (**c30**), and (**c60**) display the regional two-photon time-gated images of the kidney at different time points (5, 30, and 60 min).

**Figure 8:**
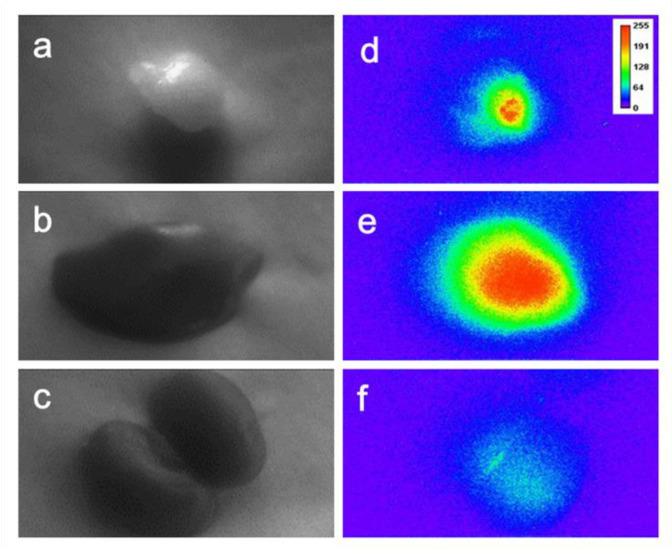
The bright-field images of the collected tissues: (**a**) tumor, (**b**) liver, and (**c**) kidney; (**d**–**f**): the sequential two-photon time-gated images of the tissues, the images were taken 75 min after injection.

In order to implement the in vivo two-photon time-gated imaging, the tumor-bearing nude mice were anesthetized; regional bright-field images of the tumors, liver, and kidneys are shown in Figure 7a–c, respectively. The mean power of laser excitation at 800 nm was 500 mW, and the beam was 12 mm in diameter, with a Gaussian distribution of the energy density. Then, the two-photon time-gated images of these tissues were scanned before the intravenous administration of Gal-DHLA-AuNCs (Figure 7a0–c0), which substantially proved the “background-free” detection of signals in this “two-photon” and “time-gated” combined method of imaging. For in vivo imaging, the Gal-DHLA-AuNCs were intravenously administered to the tumor-bearing nude mice, and the regional two-photon and time-gated images of the tumors, liver, and kidneys were scanned at different time points (5, 30, and 60 min) as the mice were anesthetized, as shown in Figure 7 (the numbers after the letters represent the time points). Afterward, the mice were sacrificed, and the tissues were collected, and the two-photon time-gated images of the tissues were scanned (Figure 8d–f), thus certifying the live imaging.

## 4. Conclusions

In summary, we employed two-photon fluorescence and time-gated imaging. Based on the benefits of two-photon fluorescence imaging and time-gated imaging, a novel approach to high-quality in vivo imaging was provided. In order to implement the investigated method, the unique characteristics of DHLA-AuNCs were studied, including the large two-photon absorption cross-section (up to 1.59 × 10^5^ GM) and prolonged fluorescence lifetime (>300 ns), which clearly distinguished them from autofluorescence. We also checked the feasibility of applying DHLA-AuNCs to biological imaging, showing their good stability and biocompatibility. Most importantly, we used bio-conjugated Gal-DHLA-AuNCs that specifically targeted hepatocellular carcinoma cells to carry out two-photon and time-gated imaging in vivo, and the successful in vivo tests demonstrated the elimination of autofluorescence signals and magnificently improved the signal-to-noise ratio.

## Data Availability

The data presented in this study are available from the corresponding author upon reasonable request.

## Supplementary Materials

The following are available online at https://www.mdpi.com/article/10.3390/ma14247744/s1, Figure S1. The arrangement of the in vivo imaging system: (A) the fixed working laser pulses, (B) a moveable object stage, and (C) the inhalation mask for anesthesia. Figure S2. The changes in the PL intensity of DHLA-AuNCs with various pH values. Figure S3. The time-course changes in the PL intensity of DHLA-AuNCs incubated in PBS containing GSH (2 m). Figure S4. The changes in the PL intensity of DHLA-AuNCs continuously exposed to a 400 nm laser for 1 h. Figure S5. DLS analysis of DHLA-AuNCs displaying a hydrodynamic diameter of 3.7 ± 0.1 nm. Figure S6. TGA curves of DHLA-AuNCs. Figure S7. Fourier Transform infrared (FTIR) spectra of conjugated probe Gal-DHLA-AuNCs and DHLA-AuNCs. Figure S8. Two-photon-induced fluorescence spectra, excited at 800 nm. (A) Rhodamine B, (B) DHLA-AuNCs.

## References

[1] Weissleder R., Pittet M.J. (2008). Imaging in the era of molecular oncology. Nature.

[2] Massoud T.F., Gambhir S. (2003). Molecular imaging in living subjects: Seeing fundamental biological processes in a new light. Genes Dev..

[3] Koo V., Hamilton P.W., Williamson K. (2016). Non-invasive in vivo imaging in small animal research. Cell. Oncol. Off. J. Int. Soc. Cell. Oncol..

[4] Wang Y., Lin X., Chen X., Chen X., Xu Z., Zhang W., Liao Q., Duan X., Wang X., Liu M. (2017). Tetherless near-infrared control of brain activity in behaving animals using fully implantable upconversion microdevices. Biomaterials.

[5] Xu Z., Song A., Wang F., Chen H. (2021). Sensitive and effective imaging of carbon monoxide in living systems with a near-infrared fluorescent probe. RSC Adv..

[6] Kim H.M., Jung C., Kim B.R., Jung S.-Y., Hong J.H., Ko Y.-G., Lee K.J., Cho B.R. (2007). Environment-Sensitive Two-Photon Probe for Intracellular Free Magnesium Ions in Live Tissue. Angew. Chem..

[7] Juvekar V., Lee H.W., Kim H.M. (2021). Two-Photon Fluorescent Probes for Detecting Enzyme Activities in Live Tissues. ACS Appl. Bio Mater..

[8] Ricard C., Arroyo E.D., He C.X., Portera-Cailliau C., Lepousez G., Canepari M., Fiole D. (2018). Two-photon probes for in vivo multicolor microscopy of the structure and signals of brain cells. Brain Struct. Funct..

[9] Helmchen F., Denk W. (2005). Deep tissue two-photon microscopy. Nat. Methods.

[10] Dahan M., Laurence T., Pinaud F., Chemla D.S., Alivisatos A.P., Sauer M., Weiss S. (2001). Time-gated biological imaging by use of colloidal quantum dots. Opt. Lett..

[11] Sakiyama M., Sugimoto H., Fujii M. (2018). Long-lived luminescence of colloidal silicon quantum dots for time-gated fluorescence imaging in the second near infrared window in biological tissue. Nanoscale.

[12] Zheng J., Zhang C., Dickson R.M. (2004). Highly Fluorescent, Water-Soluble, Size-Tunable Gold Quantum Dots. Phys. Rev. Lett..

[13] Shang L., Nienhaus G.U. (2012). Gold nanoclusters as novel optical probes for in vitro and in vivo fluorescence imaging. Biophys. Rev..

[14] Wang X., Ai A., Yu Z., Deng M., Liu W., Zhou G., Zhang W., Cao Y., Wang X. (2019). Dual-modal non-invasive imaging in vitro and in vivo monitoring degradation of PLGA scaffold based gold nanoclusters. Mater. Sci. Eng. C.

[15] Ramanujan V.K., Zhang J.-H., Biener E., Herman B. (2005). Multiphoton fluorescence lifetime contrast in deep tissue imaging: Prospects in redox imaging and disease diagnosis. J. Biomed. Opt..

[16] Al Kindi H., Mohamed A., Kajimoto S., Zhanpeisov N., Horino H., Shibata Y., Rzeznicka I.I., Fukumura H. (2018). Single bovine serum albumin molecule can hold plural blue-emissive gold nanoclusters: A quantitative study with two-photon excitation. J. Photochem. Photobiol. A Chem..

[17] Kojima N., Ikeda K., Kobayashi Y., Tsukuda T., Negishi Y., Harada G., Sugawara T., Seto M. (2012). Study of the structure and electronic state of thiolate-protected gold clusters by means of 197Au Mssbauer spectroscopy. Hyperfine Interact..

[18] Negishi Y., Nobusada K., Tsukuda T. (2005). Glutathione-protected gold clusters revisited: Bridging the gap between gold(I)-thiolate complexes and thiolate-protected gold nanocrystals. J. Am. Chem. Soc..

[19] Xu C., Webb W.W. (1996). Measurement of two-photon excitation cross sections of molecular fluorophores with data from 690 to 1050 nm. J. Opt. Soc. Am. B.

[20] Sun C., Yang H., Yuan Y., Tian X., Wang L., Guo Y., Xu L., Lei J., Gao N., Anderson G.J. (2011). Controlling Assembly of Paired Gold Clusters within Apoferritin Nanoreactor for in Vivo Kidney Targeting and Biomedical Imaging. J. Am. Chem. Soc..

[21] Jin D., Piper J.A. (2011). Time-Gated Luminescence Microscopy Allowing Direct Visual Inspection of Lanthanide-Stained Microorganisms in Background-Free Condition. Anal. Chem..

[22] Cao X., Yao C., Jiang S., Gunn J., Van Namen A.C., Bruza P., Pogue B.W. (2020). Time-gated luminescence imaging for background free in vivo tracking of single circulating tumor cells. Opt. Lett..

[23] Shang L., Dong S., Nienhaus G.U. (2011). Ultra-small fluorescent metal nanoclusters: Synthesis and biological applications. Nano Today.

[24] Wu Z., Jin R. (2010). On the Ligand’s Role in the Fluorescence of Gold Nanoclusters. Nano Lett..

[25] Li Y., Teng S., Wang M., Duan B., Huang Z. (2020). Molecular crowding-modulated fluorescence emission of gold nanoclusters: Ligand-dependent behaviors and application in improved biosensing. Sens. Actuators B Chem..

[26] Duan H., Nie S. (2007). Etching Colloidal Gold Nanocrystals with Hyperbranched and Multivalent Polymers: A New Route to Fluorescent and Water-Soluble Atomic Clusters. J. Am. Chem. Soc..

[27] Shang L., Brandholt S., Stockmar F., Trouillet V., Bruns M., Nienhaus G.U. (2014). Effect of Protein Adsorption on the Fluorescence of Ultrasmall Gold Nanoclusters. Small.

[28] Huang C.-C., Yang Z., Lee K.-H., Chang H.-T. (2007). Synthesis of Highly Fluorescent Gold Nanoparticles for Sensing Mercury(II). Angew. Chem..

[29] Jin R. (2010). Quantum sized, thiolate-protected gold nanoclusters. Nanoscale.

[30] Zhou M., Higaki T., Li Y., Zeng C., Li Q., Sfeir M.Y., Jin R. (2019). Three-Stage Evolution from Nonscalable to Scalable Optical Properties of Thiolate-Protected Gold Nanoclusters. J. Am. Chem. Soc..

[31] Le Guevel X., Spies C., Daum N., Jung G., Schneider M. (2012). Highly fluorescent silver nanoclusters stabilized by glutathione: A promising fluorescent label for bioimaging. Nano Res..

[32] Homberger M., Schmid S., Timper J., Simon U. (2012). Solid Phase Supported “Click”-Chemistry Approach for the Preparation of Water Soluble Gold Nanoparticle Dimers. J. Clust. Sci..

[33] Zheng Y., Gao S., Ying J.Y. (2010). Synthesis and Cell-Imaging Applications of Glutathione-Capped CdTe Quantum Dots. Adv. Mater..

[34] Xifeng C., Zhenzhen G., Peng M. (2018). One-pot synthesis of GSH-Capped CdTe quantum dots with excellent biocompatibility for direct cell imaging. Heliyon.

[35] Jones D.P., Carlson J.L., Samiec P.S., Sternberg P., Mody V.C., Reed R.L., Brown L.A. (1998). Glutathione measurement in human plasma. Evaluation of sample collection, storage and derivatization conditions for analysis of dansyl derivatives by HPLC. Clin. Chim. Acta.

[36] Zheng J., Zhou C., Yu M., Liu J. (2012). Different sized luminescent gold nanoparticles. Nanoscale.

[37] Fernández-Suárez M., Ting A.Y. (2008). Fluorescent probes for super-resolution imaging in living cells. Nat. Rev. Mol. Cell Biol..

[38] Zhu W., Zheng X., Huang Y., Lu Z., Ai H. (2018). Super-resolution imaging and real-time tracking lysosome in living cells by a fluorescent probe. Sci. China Ser. B Chem..

[39] Polavarapu L., Manna M., Xu Q.-H. (2010). Biocompatible glutathione capped gold clusters as one- and two-photon excitation fluorescence contrast agents for live cells imaging. Nanoscale.

[40] Valenta J., Greben M., Pramanik G., Kvakova K., Cigler P. (2021). Reversible photo- and thermal-effects on the luminescence of gold nanoclusters: Implications for nanothermometry. Phys. Chem. Chem. Phys..

[41] Patel S.A., Richards C.I., Hsiang J.-C., Dickson R.M. (2008). Water-Soluble Ag Nanoclusters Exhibit Strong Two-Photon-Induced Fluorescence. J. Am. Chem. Soc..

[42] Dou X., Chen X., Zhu H., Liu Y., Chen D., Yuan X., Yao Q., Xie J. (2019). Water-soluble metal nanoclusters: Recent advances in molecular-level exploration and biomedical applications. Dalton Trans..

[43] Ghosh S.S., Takahashi M., Thummala N.R., Parashar B., Chowdhury N.R., Chowdhury J.R. (2000). Liver-directed gene therapy: Promises, problems and prospects at the turn of the century. J. Hepatol..

[44] Kikkeri R., Lepenies B., Adibekian A., Laurino P., Seeberger P.H. (2009). In Vitro Imaging and in Vivo Liver Targeting with Carbohydrate Capped Quantum Dots. J. Am. Chem. Soc..

[45] Kundu S., Maiti S., Das T.K., Karmakar S., Roy C.N., Saha A. (2021). Synthesis of luminescent biotinylated multivalent dendrimer encapsulated quantum dots and investigation on its physico-chemical interactions with biological receptor avidin. J. Lumin..

