# Supplementary material for: Two-Photon Time-Gated In Vivo Imaging of Dihydrolipoic-Acid-Decorated Gold Nanoclusters
Source: Materials (Basel). 2021 Dec 15;14(24):7744. doi: 10.3390/ma14247744 (PMC8706569; doi:10.3390/ma14247744)
Supplement: Supplementary file 1 [file materials-14-07744-s001.zip › materials-1490343-supplementary.pdf]

materials

Supplementary Materials

Two-Photon Time-Gated In Vivo Imaging of Dihydrolipoic-Acid-Decorated Gold Nanoclusters

Ye Tian [1,*], Ming Wei [1], Lijun Wang [1], Yuankai Hong [1,*], Dan Luo [2,*] and Yinlin Sha [1,*]

[1] Department of Biophysics, Single-molecule and Nanobiology Laboratory, School of Basic Medical Sciences, Peking University, Beijing 100191, China; weiming@bjmu.edu.cn (M.W.); lijunwang@bjmu.edu.cn (L.W.)
[2] CAS Center for Excellence in Nanoscience, Beijing Key Laboratory of Micro-Nano Energy and Sensor, Beijing Institute of Nanoenergy and Nanosystems, Chinese Academy of Sciences, Beijing 100083, China
* Correspondence: ye.tian1@ge.com (Y.T.); ykhong@bjmu.edu.cn (Y.H.); luodan@binn.cas.cn (D.L); shyl@hsc.pku.edu.cn (Y.S.)

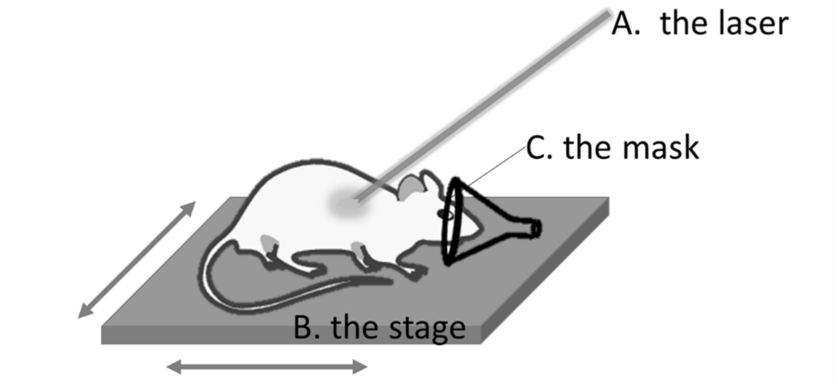

Figure S1. The arrangement of the in vivo imaging system, A) the fixed working laser pulses, B) a moveable object stage, C) the inhaling mask for anesthesia.

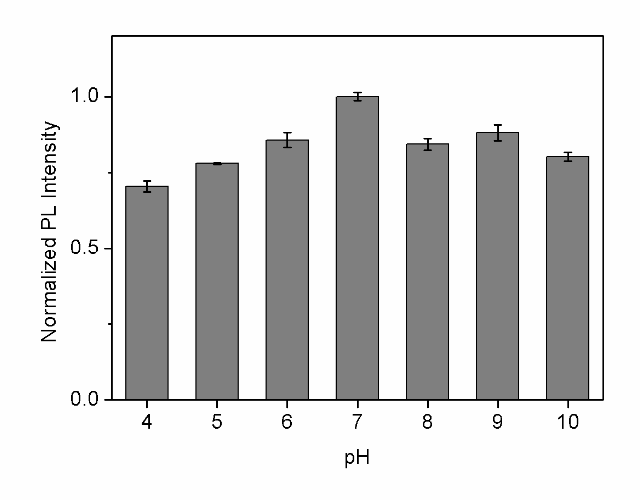

Figure S2. The PL intensity of DHLA-AuNCs changes with varied pH values.

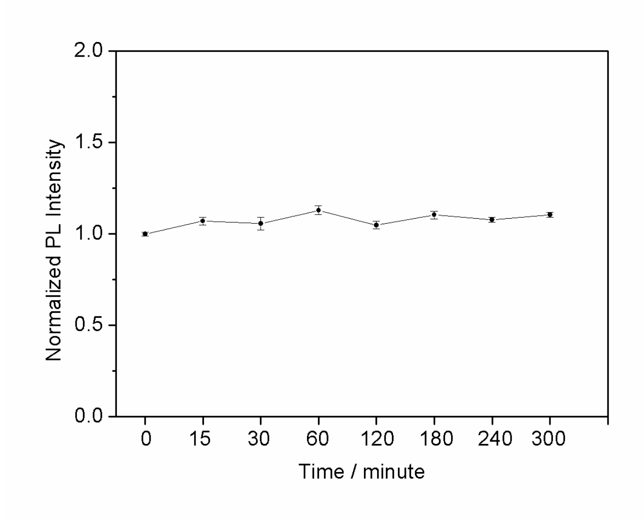

Figure S3. The time-course changes of PL intensity of DHLA-AuNCs incubated in PBS containing GSH (2 μm).

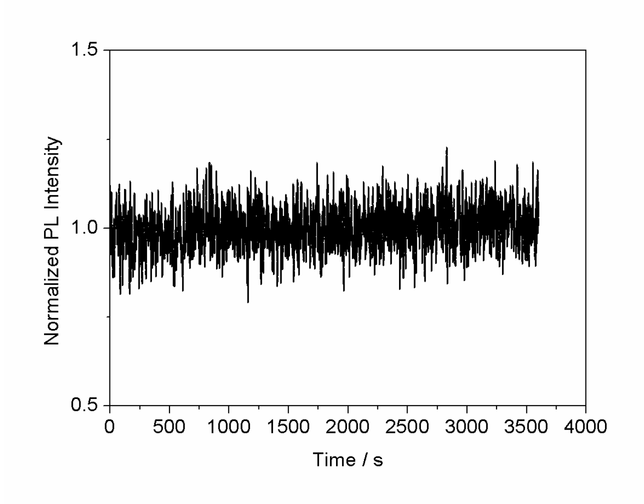

Figure S4. The change of PL intensity of DHLA-AuNCs continuously exposed to 400 nm laser for 1 h.

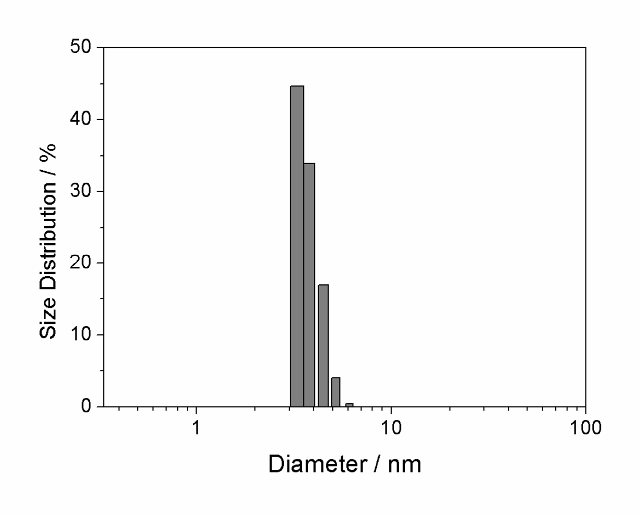

Figure S5. DLS analysis of DHLA-AuNCs displayed the hydrodynamic diameter to be (3.7±0.1) nm.

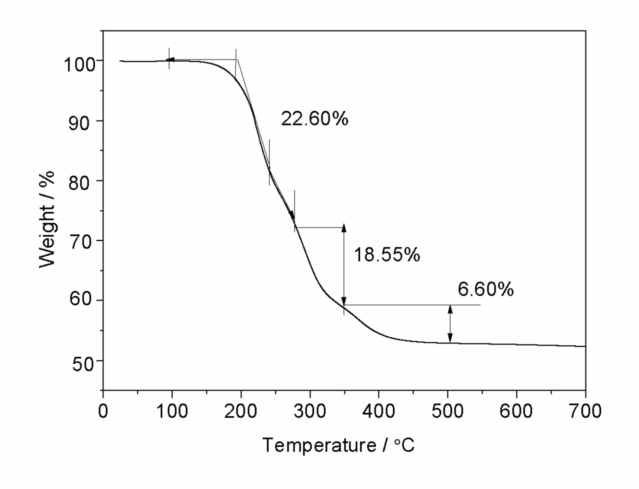

Figure S6. TGA curves of DHLA-AuNCs.

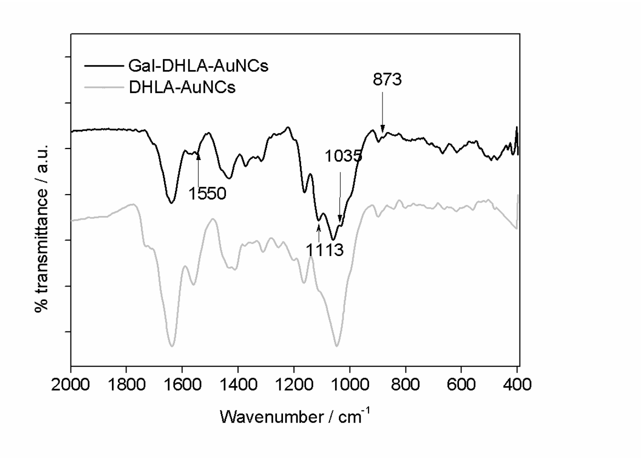

Figure S7. Fourier Transform infrared (FTIR) spectra of conjugated probe Gal-DHLA-AuNCs and DHLA-AuNCs.

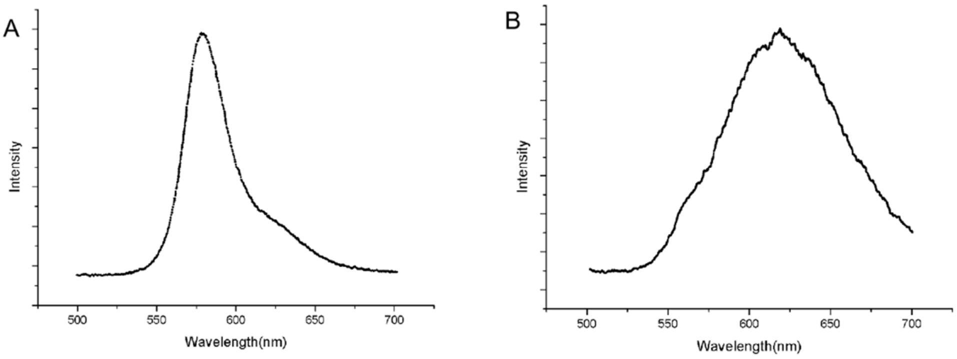

Figure S8. Two-photon-induced fluorescence spectra, excited at 800 nm. (A) Rhodamine B, (B) DHLA-AuNCs.
